# Reproducibility of the modified Neer classification defining displacement with respect to the humeral head fragment for proximal humeral fractures

**DOI:** 10.1186/s13018-020-01966-2

**Published:** 2020-09-23

**Authors:** Noboru Matsumura, Ryogo Furuhata, Takayuki Seto, Yuhei Takada, Hideyuki Shirasawa, Satoshi Oki, Yusuke Kawano, Shohei Shiono

**Affiliations:** Department of Orthopedic Surgery, Keio University School of Medicine, 35 Shinanomachi, Shinjuku-ku, Tokyo, 160-8582 Japan

**Keywords:** Proximal humeral fracture, Neer classification, Avascular necrosis, Reproducibility, Reliability, Intraobserver agreement, Interobserver agreement

## Abstract

**Background:**

Although the Neer classification is widely used for the assessment of proximal humeral fractures, its reproducibility has been challenged. The purpose of this study was to evaluate the reproducibility of the conventional Neer classification and a modified classification that defined fracture displacement with respect to the humeral head fragment.

**Methods:**

The fracture patterns in 80 cases of proximal humeral fractures were independently assessed by 6 observers. The cases were grouped according to the conventional Neer classification using radiographs followed by computed tomography (CT) scans by each examiner twice with a 1-month interval. The fractures were then classified with the modified Neer classification, which defined displacement of the fragment as separation of more than 1 cm or angulation of more than 45° from the humeral head fragment, twice with a 1-month interval. Kappa coefficients of the conventional and modified Neer classifications were compared.

**Results:**

The modified classification showed significantly higher intra-observer agreement than the conventional classification, both for radiographs (*P* = .028) and for CT scans (*P* = .043). Intra-observer agreement was also significantly higher for the modified classification than for the conventional classification, both for radiographs (*P* = .001) and for CT scans (*P* < .001).

**Conclusions:**

The present study showed that agreement for the Neer classification could be improved when fracture displacement was defined as separation or angulation from the humeral head. Considering vascularity to the humeral head, furthermore, the modified method might be more helpful for predicting patients’ prognosis than the conventional Neer classification.

## Introduction

Proximal humeral fractures are common injuries in the elderly [1, 2]. Whereas most of them can be treated nonoperatively, comminuted fractures often require surgical intervention [3]. Management of the fracture is determined by age, comorbidities, functional demand, and the morphology of the fracture [4]. Thus, the fractures should be classified for adequate patient selection.

For the assessment of proximal humeral fractures, several classification schemes have been used to group fracture patterns and to determine the severity of the fracture [5–9]. Of all of the classifications of proximal humeral fractures, the Neer classificatio n[8] is probably one of the most commonly used [10]. The concept of the Neer classification is dividing the proximal humerus into 4 segments (humeral head, greater tuberosity, lesser tuberosity, and humeral shaft) and classifying the proximal humeral fractures by the number of displaced segments. The purposes of this 4-segment system were to identify every type of fracture or fracture–dislocation of the proximal humerus, to document the anatomic problems and the therapeutic implications of each category, and to provide terminology that could be used worldwide to depict the pathoanatomy of each entity [9].

Similar to the other classification schemes, however, the reliability and reproducibility of the Neer classification have been challenged [11–15]. Although displacement is simply defined as the separation of more than 1 cm or angulation of more than 45°, [8] the arbitrary definition of “displacement” is a limitation of the Neer classification [10]. The judgement of the presence of separation or angulation is not always easy on 2-dimensional radiographs. Advances in computed tomography (CT) imaging technology have facilitated determining whether fractures have occurred and understanding how the fracture is displaced, and CT is now used frequently to evaluate proximal humeral fractures [16, 17]. However, its reproducibility remains in doubt even with the use of CT scans and 3-dimensional computational CT reconstructions [14, 18–21].

Since the proximal humerus is divided into 4 segments in the Neer classification, a maximum of 6 combinations of fragment displacement can occur. Prediction of ischemia of the humeral head is also an important role of the categorization of proximal humeral fractures [7]. Secondary avascular necrosis of the humeral head often occurs following fragmented proximal humeral fractures [22]. Since the Neer classification focused on posttraumatic avascular necrosis, [9] vascularity to the humeral head has to be appreciated. Thus, we proposed to add “from the humeral head fragment” to the definition of fracture displacement to simplify the classification. This study hypothesized that the reproducibility of the Neer classification would improve when fragment displacement is assessed with respect to the humeral head fragment. The purpose of this study was to evaluate intra- and inter-observer agreements of the conventional Neer classification and of the modified classification, which defined displacement of the fragment as separation of more than 1 cm or angulation of more than 45° from the humeral head fragment.

## Materials and methods

### Materials

This study was approved by the Institutional Review Board of our institution. Consecutive adult cases with proximal humeral fractures, in which both plain radiographs and CT scans had been taken during the period between 2012 and 2017, were retrospectively reviewed. This study included the cases with radiographs taken from two directions, including the standard trauma series of antero-posterior and scapular-Y views, and CT scans taken within 2 days after plain radiographs, with both axial CT scans and 3-dimensional CT reconstruction (Aquilion ONE, Canon Medical Systems Corp, Tochigi, Japan). Isolated fracture of the tuberosity, fracture on the articular surface, and pathologic fractures were excluded [10]. A total of 80 fractures (28 males, 52 females) were enrolled and evaluated. The right shoulder was involved in 37 cases, and the left shoulder was involved in 43 cases. The average age of the patients was 68.6 ± 16.1 years (range 21–94 years).

### Classification

Assessment of the fracture pattern was independently conducted by a total of 6 orthopedic surgeons, including 3 shoulder surgeons (observers 1–3) and 3 orthopedic trainees, who were young surgeons with less than 4 years of experience as orthopedic surgeons (observers 4–6). The examiners categorized the fractures into 1-part (minimal displacement), 2-part, 3-part, and 4-part fractures by the number of fracture fragments involved, [11, 21] but subcategorization of fractures was not assessed in this study. The cases were grouped according to the conventional Neer classification (conventional method) using plain radiographs, and then the CT scans for each case were assessed by each examiner. Assessment of CT scans included axial CT scans reconstructed with 1-mm-thick slices and 3-dimensional CT reconstruction. In an assessment by CT scans in this study, each examiner evaluated the fractures using both plain radiographs and CT scans. Since CT scans are taken after plain radiographs in most cases of proximal humeral fractures, assessment using both plain radiographs and CT scans could simulate the actual clinical setting. With a 1-month interval, second assessments of the fractures were performed using plain radiographs followed by CT scans. The cases were presented in a different, random order for each trial.

**Fig. 1:**
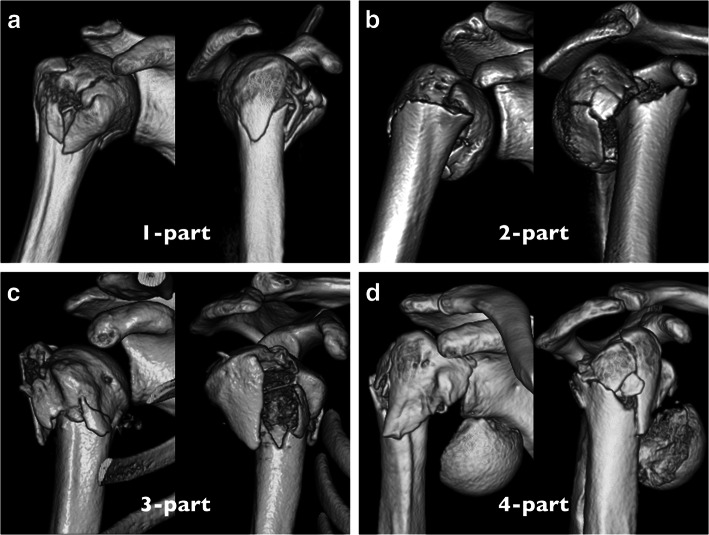
Modified Neer classification. The modified Neer classification defines displacement of the fragment as separation of more than 1 cm or angulation of more than 45° from the humeral head fragment. **a** 1-part fracture. The humeral head stays together with the other segments. **b** 2-part fracture. The humeral head with the tuberosities is rotated to the humeral shaft. **c** 3-part fracture. The humeral head is separated from the humeral shaft and greater tuberosity. **d** 4-part fracture. The humeral head is displaced from the other 3 segments

With another 1-month interval, the fractures were then classified with the modified Neer classification, which defined displacement of the fragment as separation of more than 1 cm or angulation of more than 45° from the humeral head fragment (modified method) using plain radiographs followed by CT scans (Fig. 1). Second assessments by the modified method using plain radiographs and CT scans were then performed after a 1-month interval. Thus, each observer independently grouped the fractures by radiographs and CT scans a total of 4 times with 1-month intervals between assessments, including twice by the conventional method and twice by the modified method. The order of the fractures was changed randomly before each trial.

### Statistical analyses

Statistical analyses were performed using IBM SPSS Statistics 25.0.0.0 software (IBM, Armonk, NY, USA). Data were analyzed using kappa statistics, as described by Cohen, [23] showing the agreement between two sets of observations. According to the guidelines of Landis and Koch, [24] values <0 were considered poor, 0.00–0.20 slight, 0.21–0.40 fair, 0.41–0.60 moderate, 0.61–0.80 substantial, and 0.81–1.00 almost perfect agreement. The values of the kappa statistics are reported with 95% confidence intervals.

Intra-rater agreements between 2 trials of 6 observers and inter-rater agreements of the 15 pairs of observers of both plain radiographs and CT scans of both conventional and modified methods were assessed using kappa coefficients. To assess the difference in intra-rater reliability, kappa coefficients between 2 assessments of 6 observers of both plain radiographs and CT scans were compared between the conventional and modified Neer classifications using paired *t* tests. For intra-rater reliability, kappa coefficients of the 15 pairs of inter-rater reliabilities of both plain radiographs and CT scans were compared between the conventional and modified Neer classifications using paired *t* tests. To evaluate the differences in intra- and inter-rater reliabilities between the experienced group (observers 1–3) and the inexperienced group (observers 4–6), kappa coefficients for intra-rater reliabilities (3 observers in each group) and inter-rater reliabilities (3 pairs in each group) for both the conventional and modified Neer classifications and both plain radiographs and CT scans were also compared between shoulder surgeons and orthopedic trainees using Student’s *t* tests. The significance level was set at .05 for all analyses.

## Results

### Fracture pattern

In 2 assessments by 6 observers of both plain radiographs and CT scans, 11 fractures were categorized into 1-part (mean match rate 72.0% ± 20.0%), 51 fractures into 2-part (mean match rate 70.5% ± 17.4%), 15 fractures into 3-part (mean match rate 64.7% ± 12.2%), and 3 fractures into 4-part (mean match rate 75.0% ± 14.4%) fractures using the conventional Neer classification. On the other hand, 11 fractures were categorized into 1-part (mean match rate 74.6% ± 20.5%), 46 fractures into 2-part (mean match rate 79.8% ± 16.5%), 12 fractures into 3-part (mean match rate 73.6% ± 18.8%), and 11 fractures into 4-part (mean match rate 76.5% ± 19.1%) fractures using the modified Neer classification. In the present cases, the conventional and modified Neer classifications agreed in fracture categorization for 68 fractures (85.0%) and disagreed for 12 fractures (15.0%).

### Intra-observer reliability

For plain radiographs, the intra-observer agreement for 6 observers using the conventional classification was regarded as fair in 1 (17%), moderate in 3 (50%), and substantial in 2 (33%), and the mean value was 0.563 ± 0.131 (range, 0.351–0.685). On the other hand, intra-observer agreement using the modified classification was regarded as moderate in 2 (33%), substantial in 3 (50%), and almost perfect in 1 (17%), and the mean value was 0.671 ± 0.129 (range 0.459–0.804). The modified classification showed significantly higher intra-observer agreement than the conventional classification for plain radiographs (*P* = .002) (Table 1). For plain radiographs, intra-observer agreement did not show a significant difference between experienced and inexperienced surgeons either for the conventional classification (*P* = .801) and for the modified classification (*P* = .903).

**Table 1:** Intra-observer agreements of the conventional and modified Neer classifications

Observer	Radiographs		CT scans	
	Conventional	Modified	Conventional	Modified
1	0.678 (0.541–0.815)	0.742 (0.620–0.864)	0.848 (0.746–0.950)	0.848 (0.750–0.946)
2	0.597 (0.458–0.736)	0.649 (0.512–0.786)	0.595 (0.444–0.746)	0.662 (0.523–0.801)
3	0.463 (0.304–0.621)	0.599 (0.456–0.742)	0.465 (0.306–0.624)	0.666 (0.529–0.803)
4	0.606 (0.459–0.753)	0.773 (0.642–0.893)	0.635 (0.484–0.786)	0.797 (0.679–0.915)
5	0.351 (0.194–0.508)	0.459 (0.312–0.606)	0.215 (0.076–0.354)	0.374 (0.225–0.523)
6	0.685 (0.548–0.822)	0.804 (0.690–0.918)	0.451 (0.284–0.618)	0.547 (0.386–0.708)
Mean ± SD	0.563 ± 0.131	0.671 ± 0.129	0.535 ± 0.213	0.649 ± 0.172
*P* value	*P* = .002		*P* = .013	

*SD* standard deviation

For CT scans, the intra-observer agreement for 6 observers using the conventional classification was regarded as fair in 1 (17%), moderate in 3 (50%), and substantial in 2 (33%), with a mean value of 0.535 ± 0.213 (range 215–0.848). On the other hand, that for the modified classification was regarded as fair in 1 (17%), moderate in 1 (17%), substantial in 3 (50%), and almost perfect in 1 (17%), with a mean value of 0.649 ± 0.172 (range 0.374–0.848). The modified classification showed significantly higher intra-observer agreement than the conventional classification for CT scans (*P* = .013) (Table 1). For CT scans, intra-observer agreement did not differ between experienced and inexperienced surgeons, either for the conventional classification (*P* = .289) or for the modified classification (*P* = .328).

### Inter-observer reliability

For the assessment by plain radiographs, the inter-observer agreement for the conventional classification was regarded as fair in 8 pairs (53%) and moderate in 7 pairs (47%), and the mean value was 0.398 ± 0.056 (range 0.297–0.491). On the other hand, inter-observer agreement for the modified classification was regarded as fair in 1 pair (7%), moderate in 10 pairs (67%), and substantial in 4 pairs (27%), and the mean value was 0.517 ± 0.100 (range 0.353–0.721). The modified classification showed significantly higher intra-observer agreement than the conventional classification for plain radiographs (*P* = .001) (Table 2). For plain radiographs, inter-observer agreement did not show a significant difference between experienced and inexperienced surgeons, both for the conventional classification (*P* = .518) and for the modified classification (*P* = .062).

**Table 2:** Inter-observer agreement for the conventional and modified Neer classifications

Observer	Radiographs		CT scans	
	Conventional	Modified	Conventional	Modified
1 + 2	0.385 (0.277–0.493)	0.660 (0.564–0.756)	0.530 (0.425–0.634)	0.700 (0.608–0.792)
1 + 3	0.465 (0.351–0.579)	0.606 (0.502–0.710)	0.513 (0.403–0.623)	0.645 (0.549–0.741)
1 + 4	0.351 (0.237–0.465)	0.721 (0.631–0.811)	0.455 (0.343–0.567)	0.805 (0.727–0.883)
1 + 5	0.405 (0.291–0.519)	0.492 (0.386–0.598)	0.156 (0.062–0.250)	0.452 (0.348–0.556)
1 + 6	0.335 (0.219–0.451)	0.495 (0.385–0.605)	0.250 (0.140–0.360)	0.591 (0.487–0.695)
2 + 3	0.414 (0.300–0.528)	0.516 (0.408–0.624)	0.581 (0.471–0.691)	0.614 (0.510–0.718)
2 + 4	0.370 (0.252–0.488)	0.576 (0.470–0.682)	0.499 (0.383–0.615)	0.689 (0.595–0.783)
2 + 5	0.334 (0.222–0.446)	0.402 (0.296–0.508)	0.255 (0.149–0.361)	0.339 (0.231–0.447)
2 + 6	0.395 (0.279–0.511)	0.424 (0.312–0.536)	0.357 (0.239–0.475)	0.586 (0.480–0.692)
3 + 4	0.444 (0.328–0.560)	0.609 (0.507–0.711)	0.561 (0.451–0.671)	0.621 (0.519–0.723)
3 + 5	0.419 (0.305–0.533)	0.464 (0.360–0.568)	0.232 (0.128–0.336)	0.456 (0.350–0.562)
3 + 6	0.459 (0.347–0.571)	0.475 (0.361–0.589)	0.449 (0.333–0.565)	0.477 (0.361–0.593)
4 + 5	0.297 (0.179–0.415)	0.492 (0.388–0.596)	0.292 (0.184–0.400)	0.460 (0.352–0.568)
4 + 6	0.491 (0.375–0.607)	0.471 (0.357–0.585)	0.400 (0.277–0.523)	0.611 (0.505–0.717)
5 + 6	0.342 (0.228–0.456)	0.353 (0.247–0.459)	0.385 (0.279–0.491)	0.362 (0.252–0.472)
Mean ± SD	0.398 ± 0.056	0.517 ± 0.100	0.394 ± 0.133	0.561 ± 0.131
*P* value	*P* = .001		*P* < .001	

*SD* standard deviation

For the assessment by CT scans, the inter-observer agreement for the conventional classification was regarded as poor in 1 pair (7%), fair in 7 pairs (47%), and moderate in 7 pairs (47%), and the mean value was 0.394 ± 0.133 (range 0.156–0.581). On the other hand, that for the modified classification was regarded as fair in 2 (13%), moderate in 6 (40%), substantial in 6 (40%), and almost perfect in 1 (7%), with a mean value of 0.561 ± 0.131 (range, 0.362–0.805). The modified classification showed significantly higher intra-observer agreement than the conventional classification for CT scans (*P* < .001) (Table 2). For CT scans, inter-observer agreement in the experienced group was significantly higher using the conventional classification than for the inexperienced group (*P* = .010), but no difference was found between experienced and inexperienced surgeons using the modified classification (*P* = .084).

## Discussion

The Neer classification is a 4-segment classification system for proximal humeral fractures based on the number of displaced segments [8, 9]. Although this classification is commonly used, the reproducibility of the classification was reported to not be high [11–15]. The inter-observer reliability for the Neer classification system was reported to range from fair to moderate, whereas the intra-observer reliability was somewhat better [11, 15]. In the present study, conventional methods showed moderate intra-observer agreement and fair inter-observer agreement for assessments both by plain radiographs and by CT scans with plain radiographs, and the present results were consistent with the past literature. This study showed that the reproducibility of the Neer classification improves when fragment displacement is assessed with respect to the humeral head fragment by facilitating classification in some particular fractures.

**Fig. 2:**
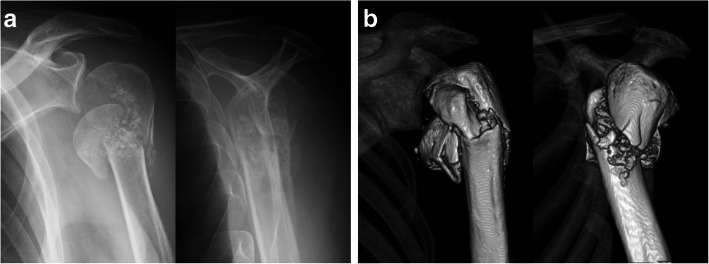
Left proximal humeral fracture of a 53-year-old man. The humeral head is displaced inferiorly with the other components staying together. The consensus of assessments is divided from 2-part fracture to 4-part fracture with the conventional Neer classification, but it is seen as a 4-part fracture with the modified classification. **a** Antero-posterior and scapular-Y views of plain radiographs. **b** Anterior and lateral views of 3-dimensional CT reconstructions

**Fig. 3:**
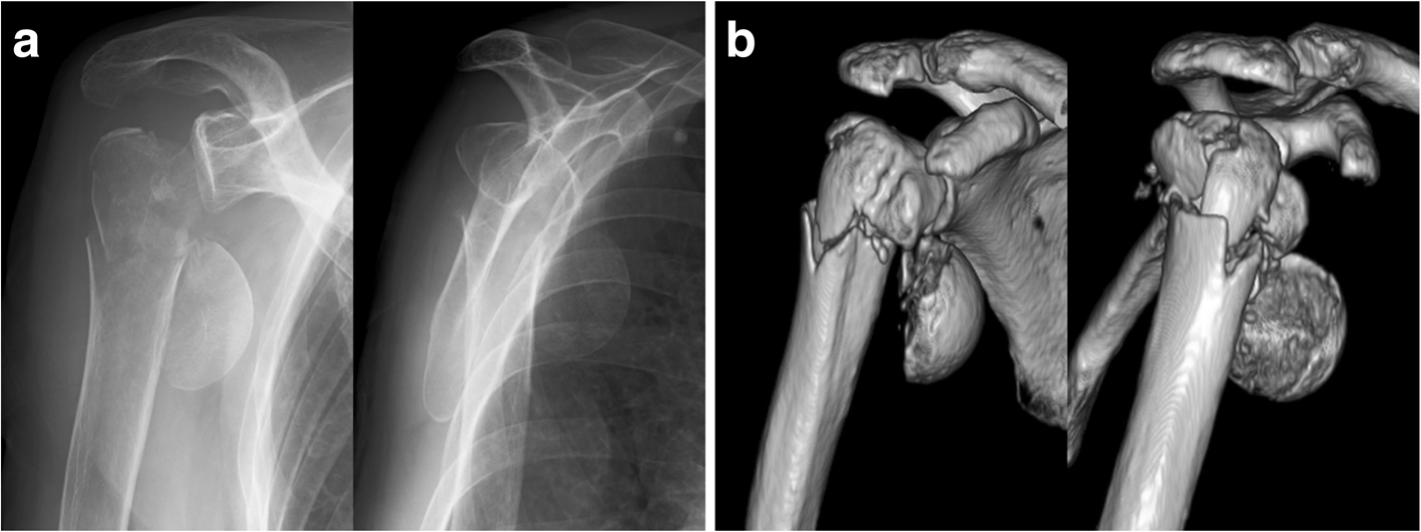
Right proximal humeral fracture of a 78-year-old woman. The humeral head is anteriorly dislocated and completely isolated from the other segments, while the fragmented tuberosities remain attached to the shaft. This fracture is often categorized as a 2-part fracture of the anatomical neck, but it is mostly grouped as 3-part or 4-part fractures according to the conventional Neer classification. Although the number of displaced segments is 2, the fracture is regarded as a 4-part fracture with the modified classification in all trials. **a** Antero-posterior and scapular-Y views of plain radiographs. **b** Anterior and lateral views of 3-dimensional CT reconstructions

The Neer classification is based on displacement of segments rather than fracture lines [9]. It defines displacement of the fracture as separation of more than 1 cm or angulation of more than 45°, [8] and disagreements in assessments in using the Neer classification arise from differences in judgement of displacement [10]. Although its definition is simple, the categorization of fractures is not always easy. Since the Neer classification divides the proximal humerus into the humeral head, greater tuberosity, lesser tuberosity, and humeral shaft, 6 combinations of fragment displacement can occur among the 4 segments. In our trials with the conventional classification, the consensus of assessments was divided from 2-part fracture to 4-part fracture when the humeral head was separated from the other 3 segments with both tuberosities staying together with the humeral shaft (Fig. 2). In these fractures, 3 of 6 combinations were displaced, whereas the other 3 combinations were not. On the other hand, all assessments were unified into 4-part fracture when fracture displacement was defined as separation or angulation from the humeral head. With the modified classification, the number of combinations of fragment displacements would be limited to 3, and it could make the categorization easier than the conventional classification. The present study showed that agreement for the classification could be improved with the modified Neer classification. The Neer classification subdivides proximal humeral fractures into 16 different subtypes in total by their fracture pattern, and a displaced anatomical neck fracture is defined as one subtype of 2-part fractures [8, 9]. However, vascularity to the humeral head for anatomical neck fracture is supposed to be the same as in the typical Neer 4-part fracture (Fig. 3 ) [9]. Considering vascularity to the humeral head, the number of detached fragments to the humeral head could be more important than the number of displaced segments. The modified method might be more helpful for predicting patients’ prognosis than the conventional Neer classification.

This study had several limitations. The selection bias of proximal humeral fractures was the first limitation. Since this study retrospectively reviewed 80 fractures in which both plain radiographs and CT scans were taken, the majority of the fractures had complex fragmentation in the proximal humerus. Although the majority of proximal humeral fractures are categorized as minimal displacement, [8] the present cases included only 11 cases (13.8%) with 1-part fractures. Unfortunately, intra- and inter-rater reliabilities were not high, even with the modified Neer classification. This selection bias can lead to low reproducibility of the classification and might have affected the results. Second, this study compared the reproducibility of the conventional Neer classification and the modified classification, and intra- and inter-observer agreements were significantly higher with the modified method. However, the efficiency of this classification was not assessed in a clinical setting. Third, this study included cases in which both plain radiographs and CT scans within 2 days after radiographs were taken. However, there might be differences in fracture displacement between radiographs in the standing position and CT scans in the supine position. Although the fracture patterns for plain radiographs did not completely correspond with those for CT scans, this study showed that the modified method could improve the reproducibility of the Neer classification both with radiographs and CT scans. The selection of observers could be another possible limitation. Although all observers were orthopedic surgeons, including 3 senior shoulder surgeons and 3 orthopedic trainees, in this study, a wide variation of kappa statistics was found among the 6 observers for intra-rater agreement and among the 15 pairs for inter-rater agreement. Assessment by radiologists or traumatologists might lead to different results. Further studies are needed to clarify the reproducibility of the modified Neer classification.

## Conclusion

The reproducibility of the conventional and modified Neer classifications was evaluated. Agreement could be improved when displacement was defined as separation or angulation from the humeral head.

## Data Availability

The datasets used and/or analyzed during the current study are available from the corresponding author on reasonable request.

## Abbreviation

CT: Computed tomography
